# 1186. Zika Virus Screening in Pregnancy in the Military Health System, 2012-2019

**DOI:** 10.1093/ofid/ofac492.1021

**Published:** 2022-12-15

**Authors:** Trevor Wellington, Jamie Fraser, Huai-Ching Kuo, Patrick Hickey, David A Lindholm

**Affiliations:** Walter Reed Army Institute of Research, Silver Spring, Maryland; Infectious Disease Clinical Research Program, Bethesda, Maryland; IDCRP, Bethesda, Maryland; Uniformed Services University, Bethesda, Maryland; Uniformed Services University of the Health Sciences, San Antonio, Texas

## Abstract

**Background:**

Zika virus (ZIKV) is an emerging arthropod-borne viral disease capable of causing fetal complications in pregnant patients exposed to the virus. Pregnant women are at risk of contracting ZIKV when they (or their partners) travel to areas with known transmission. Currently, asymptomatic prenatal screening for ZIKV is not routinely recommended, regardless of travel history, though rates of birth defects are similar between symptomatic and asymptomatic women. We sought to evaluate the rate of true positivity among pregnant patients coded for ZIKV in the Military Health System (MHS).

**Methods:**

KAPOS (Deployment and Travel Health: Knowledge, Attitudes, Practices, and Outcomes Study) is a multi-cohort study evaluating the burden of travel-associated diseases in the MHS. The MHS Data Repository was searched for International Classification of Diseases (ICD)-10 codes for ZIKV infection in military beneficiaries receiving care during fiscal years 2012-2019. Subjects then underwent chart review for history of travel, diagnostic confirmation, pregnancy status, and pregnancy complications.

**Results:**

230 unique subjects coded for ZIKV were identified. 124 subjects underwent chart review for diagnostic confirmation, of which 58 (47%) were pregnant. Of pregnant subjects captured for ZIKV, 41 (71%) were ruled out by laboratory evaluation, while 7 (12%) subjects were determined to have suspected, probable, or confirmed ZIKV. All subjects but one were asymptomatic. Travel locations associated with ZIKV in pregnancy were Puerto Rico (n=2), Guyana (n=2), and Brazil (n=1). Two subjects reported only partner travel to ZIKV regions (Cuba, Guyana). There were no documented cases of microcephaly, fetal loss, or preterm birth.

**Conclusion:**

ZIKV infection in pregnancy can result in fetal malformations, though the role of laboratory screening in pregnant patients remains uncertain. While a majority of pregnant subjects coded for ZIKV were excluded by laboratory evaluation upon review, several cases of ZIKV were identified through screening, particularly in those with travel (or partner travel) to endemic regions. This study provides further insight into the importance of prenatal screening for ZIKV in pregnancy, particularly among those with personal and/or partner travel to endemic regions.

**Disclosures:**

**All Authors**: No reported disclosures.

